# Optimizing Lung‐molGPA‐Directed Therapy for EGFR/ALK/ROS1‐Wildtype Non‐Small Cell Lung Cancer Brain Metastases: Survival Outcomes Analysis

**DOI:** 10.1002/mco2.71054

**Published:** 2026-10-11

**Authors:** Bin Feng, Dingyi Yang, Xin Yang, Huanli Luo, Tian Wang, Liyuan Chen, Liang Tan, Liang Du, Yongzhong Wu, Wei Zhou, Ying Wang, Fu Jin

**Affiliations:** ^1^ Department of Radiation Oncology Chongqing University Cancer Hospital Chongqing P. R. China

**Keywords:** brain metastases, immunotherapy, lung‐molGPA, non‐small cell lung cancer, prognostic stratification, radiotherapy

## Abstract

Patients with non‐small cell lung cancer (NSCLC) and brain metastases whose tumors lack EGFR, ALK, or ROS1 alterations have heterogeneous outcomes and limited evidence to guide treatment intensity. We assessed whether Lung‐molGPA could stratify prognosis and inform treatment selection in this population. This retrospective two‐center cohort included 811 patients with EGFR/ALK/ROS1‐wild‐type NSCLC treated between September 2013 and August 2022. Overall survival (OS) was evaluated using Kaplan–Meier methods and center‐stratified multivariable Cox regression across three Lung‐molGPA strata and treatment modalities. Median OS was 6.9 months (95% confidence interval [CI], 6.4–7.4) overall and 4.81 (4.20–5.30), 7.65 (6.73–8.40), and 16.00 (13.40–22.90) months in the Lung‐molGPA 0–1.0, 1.5–2.0, and 2.5–3.0 groups, respectively (*p* < 0.001). Compared with the 0–1.0 group, adjusted mortality was lower in the 1.5–2.0 (hazard ratio [HR], 0.68; 95% CI, 0.58–0.80) and 2.5–3.0 groups (HR, 0.39; 95% CI, 0.30–0.50). Chemotherapy, immunotherapy, antiangiogenic therapy, and brain‐ and chest‐directed local therapy were independently associated with lower mortality, although their associations varied across prognostic strata and treatment contexts. Lung‐molGPA provides clinically meaningful prognostic separation and may help tailor treatment intensity for EGFR/ALK/ROS1‐wild‐type NSCLC with brain metastases.

## Graphical abstract

Currently, there are no established guidelines providing clear treatment strategies for lung cancer patients with brain metastases; this study evaluates the prognostic and therapeutic utility of the Lung‐molGPA model in 811 EGFR/ALK/ROS1‐wildtype non‐small cell lung cancer patients with brain metastases from two tertiary centers in Southwest China between September 2013 and August 2022, analyzing survival outcomes across low (0–1.0), intermediate (1.5–2.0), and high (2.5–3.0) Lung‐molGPA strata using systemic and local therapies, thereby contributing valuable clinical insights for personalized treatment planning.

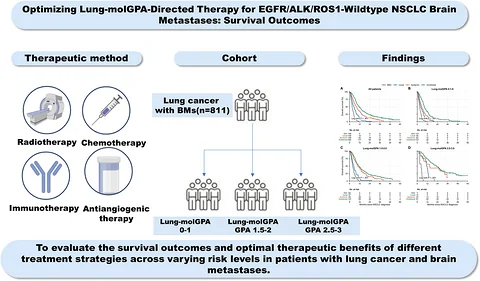

## 1 Introduction

Lung cancer remains the leading cause of cancer‐related mortality worldwide [1]. Brain metastases (BMs) are one of its most frequent and debilitating complications, with approximately 10%–20% of patients presenting with BMs at initial diagnosis [1, 2, 3]. The prognosis of non‐small cell lung cancer (NSCLC) patients with BMs has historically been dismal, typically characterized by a median overall survival (mOS) measured in mere months. This bleak outlook, however, has been transformed by significant progress across multiple therapeutic domains, including advances in diagnostic imaging techniques, innovative radiotherapy approaches, and the development of novel targeted therapies and immunotherapeutic agents. These collective breakthroughs have substantially broadened the spectrum of available treatment options, thereby creating an imperative for more sophisticated patient stratification systems to inform personalized therapeutic strategies.

To address the growing complexity of therapeutic decision‐making, several prognostic models have been developed, among which the Graded Prognostic Assessment (GPA) and its refined version, the Lung‐molGPA, have gained wide acceptance [3, 4]. These models integrate clinical and molecular factors—such as age, performance status, number of brain metastases, presence of extracranial disease, and driver mutations (e.g., EGFR, ALK, and ROS1)—to provide more personalized survival predictions [5]. Importantly, Lung‐molGPA enables clinicians to classify patients into distinct prognostic strata, each associated with significantly different mOS, thereby serving as a useful tool for clinical trial design and therapeutic planning [4, 6].

The clinical utility of the Lung‐molGPA scoring system, while validated through numerous retrospective and prospective investigations, presents several unresolved challenges that warrant further examination [4, 7]. A significant limitation lies in the lack of large‐scale validation of the Lung‐molGPA model in Chinese real‐world populations, particularly in understudied regions like Southwest China. Additionally, the prognostic value of Lung‐molGPA remains under‐characterized in EGFR/ALK/ROS1 wild‐type NSCLC patients with brain metastases, a clinically distinct subgroup that warrants further investigation. Such analyses are crucial for identifying potential selection biases in therapeutic decision‐making and uncovering opportunities for optimized treatment approaches in specific prognostic subgroups [8]. Furthermore, while the established correlation between Lung‐molGPA scores and survival outcomes remains undisputed, the proportional impact of therapeutic interventions versus other clinical variables on this observed survival benefit requires clarification [9]. This fundamental uncertainty underscores a pivotal clinical question: whether the Lung‐molGPA system serves merely as a passive prognostic classifier or possesses the capacity to actively guide therapeutic strategy formulation [10, 11].

Crucially, the clinical trajectory of NSCLC patients with BMs diverges significantly based on their molecular profile. Patients harboring actionable driver mutations (such as EGFR, ALK, or ROS1) often exhibit a distinct natural history; while they may have a high propensity for central nervous system (CNS) dissemination, their OS has been dramatically extended due to the advent of highly CNS‐penetrant tyrosine kinase inhibitors (TKIs) [12]. In stark contrast, the driver‐negative (wild‐type) population faces a generally poorer and more heterogeneous prognosis. Without the option of highly effective targeted therapies, management for wild‐type patients relies heavily on a complex integration of cytotoxic chemotherapy, immune checkpoint inhibitors, antiangiogenic agents, and localized interventions (radiotherapy or surgery). This fundamental difference in therapeutic landscape underscores the necessity of evaluating prognostic tools and treatment strategies specifically within the wild‐type cohort, where clinical decision‐making is more challenging and survival outcomes vary widely [13, 14].

In light of these considerations, we conducted a retrospective multicenter cohort study of 811 NSCLC patients with brain metastases, treated at two cancer centers over a 9‐year period. Our study aimed to (1) characterize the baseline demographic and treatment patterns across Lung‐molGPA‐defined prognostic groups; (2) identify clinical and therapeutic predictors of OS using multivariable Cox regression analysis; and (3) assess how survival outcomes vary with treatment modality within different Lung‐molGPA strata. By integrating prognostic modeling with real‐world treatment data, this study provides a refined understanding of the clinical utility of Lung‐molGPA in guiding management and improving outcomes in NSCLC patients with brain metastases.

## 2 Results

### 2.1 Study Population

This retrospective study was designed to assess the prognostic and therapeutic implications of Lung‐molGPA stratification in NSCLC patients with BMs lacking actionable mutations. We first identified eligible patients from two institutional databases between 2013 and August 2022, excluding those with EGFR/ALK/ROS1 alterations or incomplete clinical data (Figure 1). Clinical OS, the primary endpoint, was defined as the time from initial pathological diagnosis of NSCLC to death, with censoring at the last follow‐up or data cutoff, derived from electronic health record‐derived composite mortality data. Baseline demographics, tumor characteristics, and treatment details were extracted and categorized by Lung‐molGPA scores (0–1.0, 1.5–2.0, and 2.5–3.0).

**FIGURE 1:**
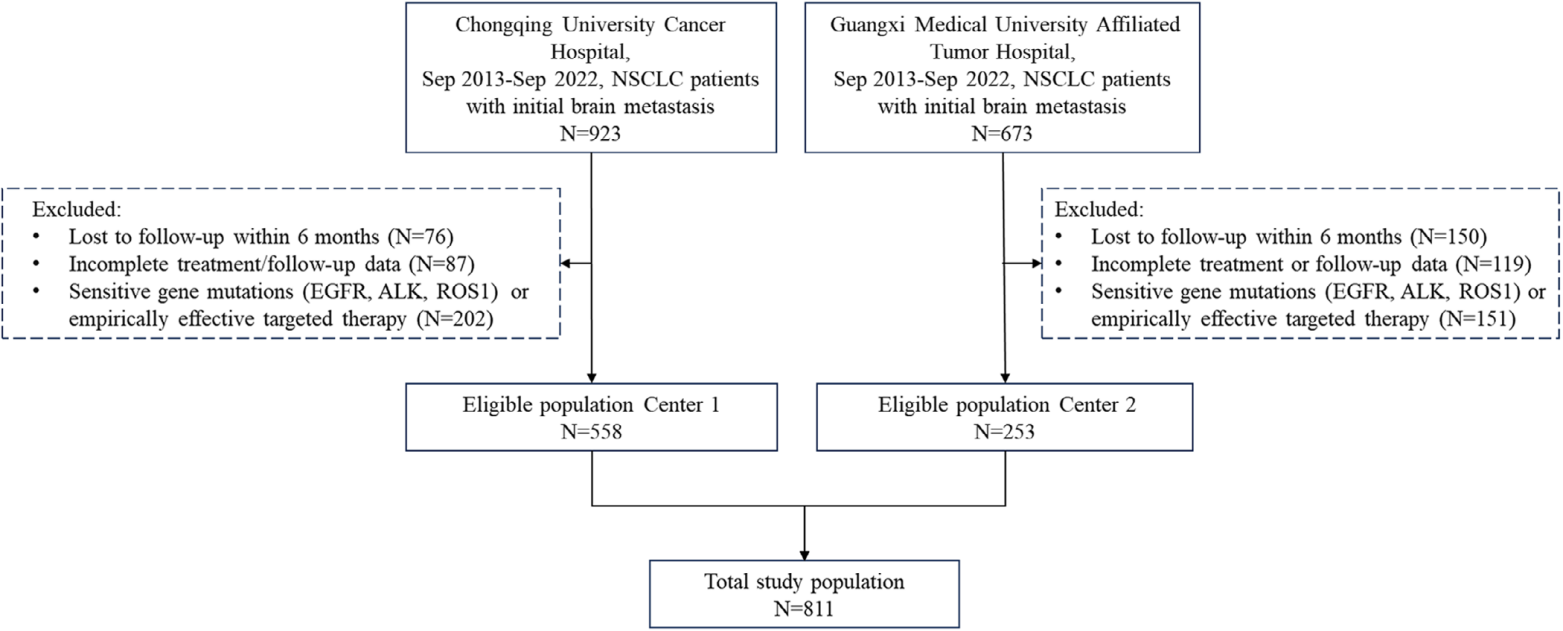
Inclusion and exclusion process for primary brain metastasis in non‐small cell lung cancer (NSCLC): A Dual‐Center Cohort.

### 2.2 Database Establishment and Analysis of Baseline Characteristics

Table 1 summarizes the baseline characteristics and treatment distributions among 811 patients with NSCLC and BMs, stratified by Lung‐molGPA scores (0–1.0, 1.5–2.0, and 2.5–3.0). The cohort was predominantly male (67%, *n* = 541), with females (33%, *n* = 270) more represented in the highest Lung‐molGPA group (2.5–3.0; 90 females vs 33 males; *p* < 0.001). Most patients were aged <70 years (84%, *n* = 683), with only 1 patient aged ≥ 70 years in the highest Lung‐molGPA group. Intracranial disease burden showed 54% (*n* = 441) with 1–4 BMs and 46% (*n* = 370) with > 4 BMs, with higher Lung‐molGPA groups having fewer lesions (*p* < 0.001). KPS of 90–100 was concentrated in the highest Lung‐molGPA group (*n* = 50), while KPS ≤ 70 was absent in this group (*p* < 0.001). Smoking history (53%, *n* = 433) and histological subtypes (adenocarcinoma, 68%; squamous cell carcinoma, 15%; other, 17%) showed no significant differences across Lung‐molGPA groups (*p* = 0.650 and *p* = 0.089, respectively). Treatment distributions varied significantly (*p* < .001), with higher Lung‐molGPA groups receiving more chemotherapy (≥ 4 cycles), immunotherapy, antiangiogenic therapy, brain radiotherapy/surgery, chest radiotherapy/surgery, and combined local‐systemic therapy, while best supportive care was more common in lower Lung‐molGPA groups.

**TABLE 1:** Baseline characteristics and treatment distribution in 811 patients with non‐small cell lung cancer (NSCLC) and brain metastases, stratified by Lung‐molGPA scores.

	Total	Lung‐molGPA 0–1	Lung‐molGPA 1.5‐2	Lung‐molGPA 2.5‐3	*p*‐Value
Gender					
Male	541 (67%)	268 (33%)	240 (30%)	33 (4%)	<0.001
Female	270 (33%)	102 (13%)	78 (10%)	90 (11%)	
Age					
<70	683 (84%)	277 (34%)	284 (35%)	122 (15%)	<0.001
≥70	128 (16%)	93 (11%)	34 (4%)	1 (0%)	
Number of BMs					
1‐4	441 (54%)	131 (16%)	201 (25%)	109 (13%)	<0.001
>4	370 (46%)	239 (29%)	117 (14%)	14 (2%)	
KPS					
90–100	121 (15%)	2 (0%)	69 (9%)	50 (6%)	<0.001
80	572 (71%)	286 (35%)	213 (26%)	73 (9%)	
≤70	118 (14%)	82 (10%)	36 (4%)	0 (0%)	
Smoking history					
Yes	433 (53%)	201 (25%)	171 (21%)	61 (8%)	0.650
No	378 (47%)	169 (21%)	147 (18%)	62 (8%)	
Pathological					
Adenocarcinoma	550 (68%)	248 (31%)	215 (27%)	87 (11%)	0.089
Squamous cell carcinoma	127 (15%)	52 (6%)	50 (6%)	25 (3%)	
Other	134 (17%)	70 (9%)	53 (7%)	11 (1%)	
Chemotherapy cycles					
1‐3	230 (28%)	101 (12%)	94 (12%)	35 (4%)	<0.001
≥4	256 (32%)	78 (10%)	111 (14%)	67 (8%)	
Absent	325 (40%)	191 (24%)	113 (14%)	21 (3%)	
Immunotherapy					
Present	103 (13%)	38 (5%)	42 (5%)	23 (3%)	0.049
Absent	708 (87%)	332 (41%)	276 (34%)	100 (12%)	
Antiangiogenic therapy					
Present	137 (17%)	42 (5%)	60 (7%)	35 (4%)	<0.001
Absent	674 (83%)	328 (40%)	258 (32%)	88 (11%)	
Brain radiotherapy/surgery					
Present	408 (50%)	155 (19%)	172 (21%)	81 (10%)	<0.001
Absent	403 (50%)	215 (27%)	146 (18%)	42 (5%)	
Chest radiotherapy/surgery					
Present	141 (17%)	36 (4%)	54 (7%)	51 (6%)	<0.001
Absent	670 (83%)	334 (41%)	264 (33%)	72 (9%)	
Treatment strategy					
Best supportive care	198 (25%)	131 (16%)	60 (7%)	7 (1%)	<0.001
Local therapy	109 (13%)	50 (6%)	47 (6%)	12 (1%)	
Systemic therapy	149 (18%)	62 (8%)	64 (8%)	23 (3%)	
Local+Systemic therapy	355 (44%)	127 (16%)	147 (18%)	81 (10%)	
PD‐L1					
Unknown	685(84%)	319(39%)	267(33%)	99(12%)	0.004
<1%	79(10%)	41(5%)	28(4%)	10(1%)	
≥1%	47(6%)	10(1%)	23(3%)	14(2%)	

**Abbreviations**: Best Supportive Care: Palliative care only, without active anticancer treatment.

Local Therapy: Brain or chest radiotherapy/surgery without systemic treatment.

Systemic Therapy: Chemotherapy, immunotherapy, or antiangiogenic therapy, without local treatments.

Local+Systemic Therapy: Combination of brain or chest RT/surgery with systemic therapy.

PD‐L1 status was unavailable in 685 of 811 patients (84%). Among the 126 patients with available results, 79 had PD‐L1 expression <1% and 47 had expression ≥1%. The comparison across Lung‐molGPA strata included all three categories—unknown, <1%, and ≥1%—using Pearson's chi‐square test (*p* = 0.004). PD‐L1 was summarized descriptively and was not included in the multivariable survival models.

With regard to systemic treatment, 28% (*n* = 230) of patients received 1–3 cycles of chemotherapy, 32% (*n* = 256) received four or more cycles, and 40% (*n* = 325) received no chemotherapy. Higher Lung‐molGPA categories had a greater proportion of patients receiving multiple cycles. Immunotherapy was administered to 13% (*n* = 103) of patients, with a higher proportion observed in the Lung‐molGPA 2.5–3.0 group (*n* = 23). Antiangiogenic therapy was utilized in 17% (*n* = 137) of the cohort, most frequently in higher Lung‐molGPA categories. Local therapies were frequently employed, including brain radiotherapy or surgery in 50% (*n* = 408) and chest‐directed interventions in 17% (*n* = 141) of patients. Utilization of these modalities was more common in patients with higher Lung‐molGPA scores. Finally, treatment strategies were heterogeneous across the cohort: 25% (*n* = 198) received best supportive care, 13% (*n* = 109) underwent local therapy only, 18% (*n* = 149) received systemic therapy only, and 44% (*n* = 355) received combined local and systemic therapy. Notably, patients with Lung‐molGPA 2.5–3.0 were more likely to receive the most intensive multimodal approach (*n* = 81), whereas those with Lung‐molGPA 0–1.0 were most frequently managed with supportive care alone (*n* = 131).

### 2.3 mOS by Lung‐molGPA Score in NSCLC Patients With Brain Metastases

All with EGFR/ALK/ROS1‐wildtype status, mOS was 6.9 months (95% confidence interval [CI]: 6.4–7.4). Group 1 (Lung‐molGPA 0–1.0, *n* = 370) had an mOS of 4.81 months (95% CI: 4.20–5.30); Group 2 (Lung‐molGPA 1.5–2.0, *n* = 318) had an mOS of 7.65 months (95% CI: 6.73–8.40); Group 3 (Lung‐molGPA 2.5–3.0, *n* = 123) had an mOS of 16.00 months (95% CI: 13.40–22.90). The log‐rank test revealed significant differences in survival across the three groups (*p* < 0.001), indicating that higher Lung‐molGPA scores were associated with improved survival outcomes in this population.

### 2.4 Cox Regression Analysis of Survival Factors

Table 2 summarizes the univariable and multivariable Cox regression analyses. Using the low Lung‐molGPA group (0–1.0) as the reference, the intermediate‐score group was associated with lower mortality (adjusted hazard ratio [HR], 0.68; 95% CI, 0.58–0.80; *p* < 0.001), as was the high‐score group (adjusted HR, 0.39; 95% CI, 0.30–0.50; *p* < 0.001). Smoking history was associated with higher mortality (adjusted HR, 1.27; 95% CI, 1.10–1.47; *p* = 0.001). Squamous‐cell carcinoma was not independently associated with OS (adjusted HR, 1.21; 95% CI, 0.98–1.48; *p* = 0.072), whereas other NSCLC histology was associated with higher mortality (adjusted HR, 1.35; 95% CI, 1.10–1.66; *p* = 0.005). Compared with no chemotherapy, 1–3 cycles (adjusted HR, 0.58; 95% CI, 0.48–0.69) and ≥4 cycles (adjusted HR, 0.23; 95% CI, 0.19–0.29) were associated with lower mortality (both *p* < 0.001). Immunotherapy (adjusted HR, 0.64; 95% CI, 0.50–0.82), antiangiogenic therapy (adjusted HR, 0.68; 95% CI, 0.54–0.85), brain‐directed local therapy (adjusted HR, 0.66; 95% CI, 0.57–0.77), and chest‐directed local therapy (adjusted HR, 0.67; 95% CI, 0.54–0.83) were also associated with lower mortality.

**TABLE 2:** Univariable and multivariable Cox regression analyses of overall survival.

	Univariate analysis		Multivariate analyses	
	**HR (95% CI)**	***p*‐Value**	**HR (95% CI)**	***p*‐Value**
Lung‐molGPA group				
0.0–1.0	1.00 (reference)			
1.5–2.0	0.57 (0.49–0.66)	<0.001***	0.68 (0.58–0.80)	<0.001***
2.5–3.0	0.29 (0.23–0.36)	<0.001***	0.39 (0.30–0.50)	<0.001***
Smoking history				
Absent	1.00 (reference)		1.00 (reference)	
Present	1.27 (1.20–1.46)	0.001**	1.27 (1.10–1.47)	0.001**
Pathological				
Adenocarcinoma	1.00 (reference)		1.00 (reference)	
Squamous cell carcinoma	1.35 (1.11–1.65)	0.003**	1.21 (0.98–1.48)	0.072
Other	1.77 (1.46–2.14)	<0.001***	1.35 (1.10–1.66)	0.005**
Chemotherapy cycles				
Absent	1.00 (reference)			
1–3	0.49 (0.41–0.58)	<0.001***	0.58 (0.48–0.69)	<0.001***
≥4	0.16 (0.13–0.20)	<0.001***	0.23 (0.19–0.29)	<0.001***
Immunotherapy				
Absent	1.00 (reference)		1.00 (reference)	
Present	0.42 (0.33–0.53)	<0.001***	0.64 (0.50–0.82)	<0.001***
Antiangiogenic therapy				
Absent	1.00 (reference)		1.00 (reference)	
Present	0.38 (0.31–0.46)	<0.001***	0.68 (0.54–0.85)	0.001**
Brain radiotherapy/surgery				
Absent	1.00 (reference)		1.00 (reference)	
Present	0.54 (0.46–0.62)	<0.001***	0.66 (0.57–0.77)	<0.001***
Chest radiotherapy/surgery				
Absent	1.00 (reference)		1.00 (reference)	
Present	0.41 (0.33–0.50)	<0.001***	0.67 (0.54–0.83)	<0.001***

HR = hazard ratio; CI = confidence interval;

*: p<0.05, **: *p* < 0.01, ***: *p* < 0.001;

### 2.5 Prognostic Impact of Lung‐molGPA Scores

Figure 2 presents the adjusted association of high versus low Lung‐molGPA with OS in the overall cohort and prespecified clinical subgroups. Among all 811 patients, including 769 deaths, the high‐score group was associated with lower mortality than the low‐score group (adjusted HR, 0.39; 95% CI, 0.30–0.50; *p* < 0.001). HRs were consistently below 1 across the displayed subgroups. Global score‐by‐subgroup interactions were not significant for smoking history (*p* = 0.565), pathology (*p* = 0.714), chemotherapy cycles (*p* = 0.280), immunotherapy (*p* = 0.127), or antiangiogenic therapy (*p* = 0.701). Evidence of heterogeneity was observed for brain‐directed local therapy (*p* for interaction<0.001) and chest‐directed local therapy (*p* for interaction = 0.039). These interaction results indicate variation in the prognostic association of Lung‐molGPA across subgroups.

**FIGURE 2:**
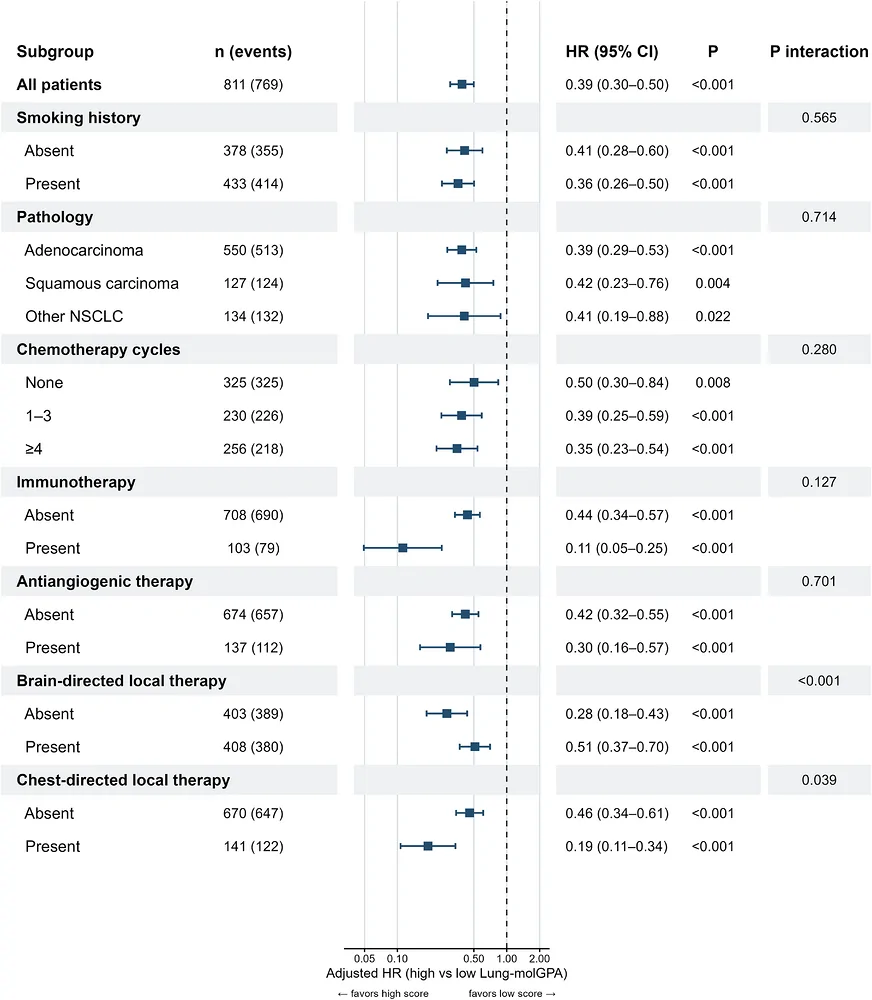
Forest plot analysis of hazard ratios for Lung‐molGPA 0–1 (Low) and 2.5–3 (High) treatment efficacy and risk factors.

To further explore the significant interaction between brain radiotherapy and Lung‐molGPA scores, patients were stratified into four subgroups according to brain radiotherapy status and Lung‐molGPA risk (0–1 vs. 2.5–3). KM survival curves were generated for each subgroup, and survival analyses using Cox proportional hazards modeling demonstrated differential survival benefits of brain radiotherapy across Lung‐molGPA strata. Among patients receiving brain‐directed local therapy, those with a Lung‐molGPA score of 2.5–3.0 had longer OS than those with a score of 0–1.0 (reference group; HR, 0.40; 95% CI, 0.30–0.53; *p* < 0.001, Table S1). In contrast, for patients with a Lung‐molGPA score of 0–1, the absence of brain radiotherapy was associated with a significantly increased risk of death (HR = 2.75, 95% CI: 2.20–3.43, p < 0.001). However, the Kaplan–Meier curves indicate that the overall prognosis of the 0–1 group remained poor regardless of treatment, suggesting limited clinical benefit from brain radiotherapy in this subgroup. Differences were compared using the log‐rank test (Figure 3 and Table S1).

As illustrated in Figure 3, patients with low Lung‐molGPA scores (0–1) who did not receive brain radiotherapy exhibited the poorest prognosis, with a steep decline in survival and no patients surviving beyond 24 months. In contrast, patients with high Lung‐molGPA scores (2.5–3) showed significantly improved survival regardless of brain radiotherapy status.

**FIGURE 3:**
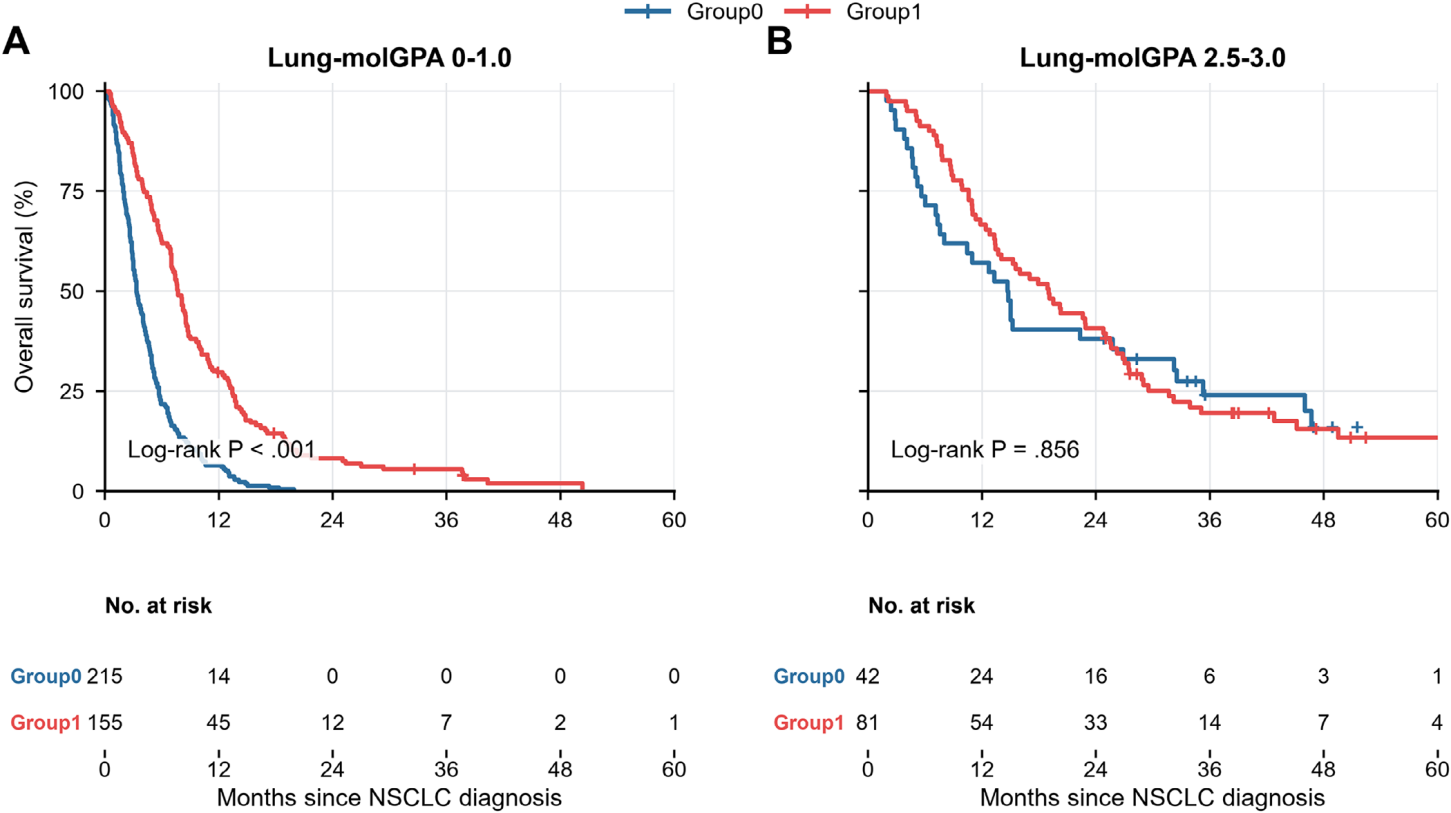
Kaplan–Meier survival curves stratified by Lung‐molGPA score (0–1 vs. 2.5–3) and brain radiotherapy status. Patients were divided into four subgroups: Group 1 represented patients who received brain radiotherapy, while Group 0 included those who did not undergo brain‐directed radiotherapy.

Pairwise comparisons further confirmed these differences, with all between‐group comparisons yielding statistically significant differences (*p* < 0.005), except between the two high‐Lung‐molGPA groups (with and without brain radiotherapy), which showed no significant difference (*p* = 0.86). This suggests that in patients with favorable prognostic features (i.e., high Lung‐molGPA scores), brain radiotherapy may not confer additional survival benefit, whereas in poor‐prognosis patients (Lung‐molGPA 0–1), absence of brain radiotherapy is associated with dramatically worse outcomes.

### 2.6 Survival Outcomes by Treatment Modality

Figure 4 shows Kaplan‐Meier estimates of OS according to treatment modality in the overall cohort and within each Lung‐molGPA stratum. In the overall cohort, OS differed significantly across treatment modalities (global log‐rank *p* < .001), with mOS increasing from 3.1 months in patients receiving best supportive care to 5.2 months with local therapy alone, 8.2 months with systemic therapy alone, and 11.4 months with combined systemic and local therapy. Similar separation was observed after stratification by Lung‐molGPA score. In the Lung‐molGPA 0–1.0 stratum, mOS was 2.9, 4.0, 5.2, and 8.4 months for best supportive care, local therapy, systemic therapy, and combined therapy, respectively (global log‐rank *p* < 0.001). Corresponding mOS values were 3.6, 5.1, 9.8, and 11.4 months in the Lung‐molGPA 1.5‐2.0 stratum (global log‐rank *p* < 0.001), and 4.6, 11.0, 14.8, and 22.8 months in the Lung‐molGPA 2.5‐3.0 stratum (global log‐rank *p* < 0.001). These findings indicate that treatment modality was associated with OS across prognostic strata, with combined systemic and local therapy showing the longest mOS in each Lung‐molGPA group.

**FIGURE 4:**
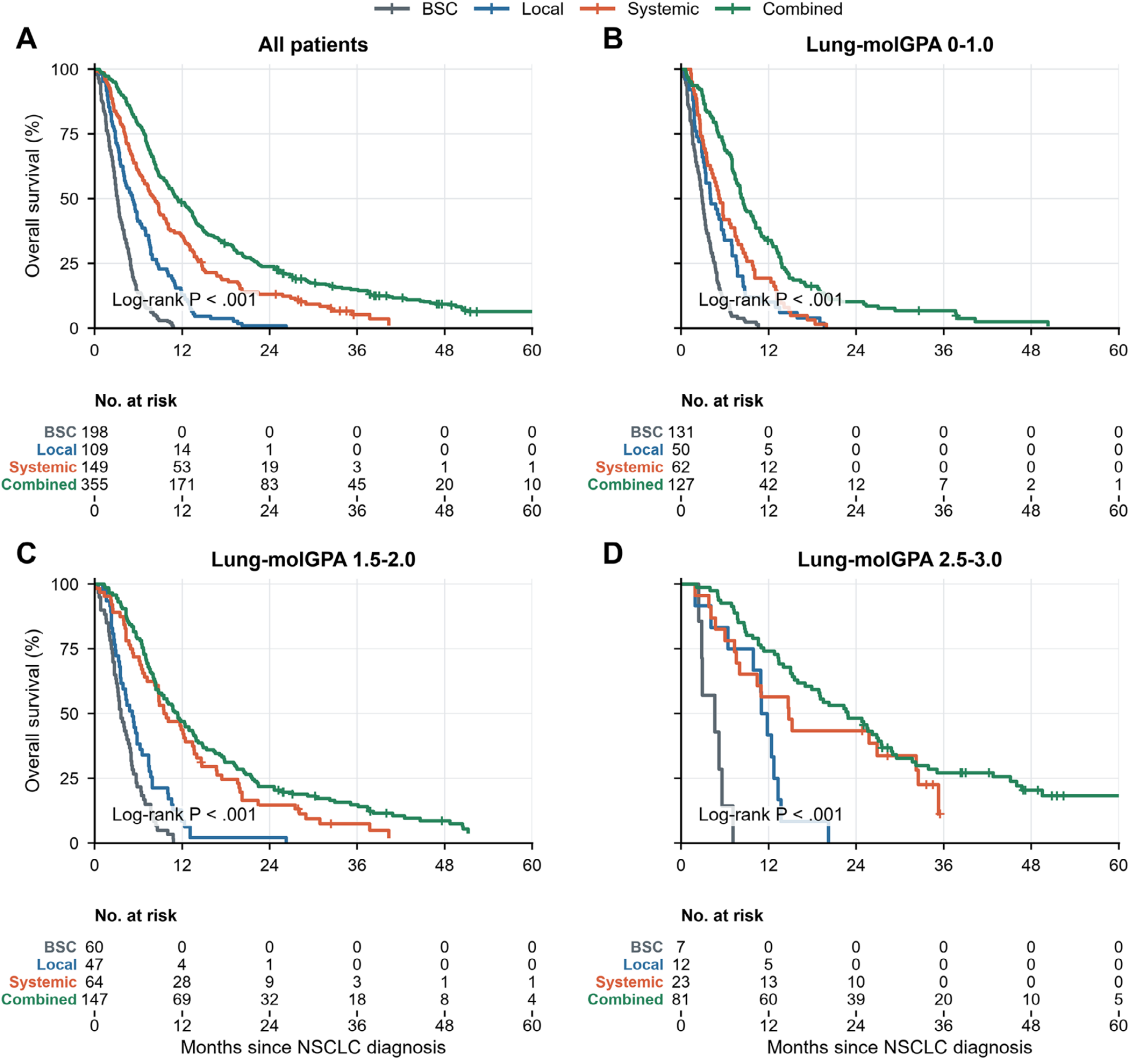
Kaplan‐Meier estimates of overall survival by treatment modality and Lung‐molGPA score. (A) Overall cohort. (B–D) Lung‐molGPA 0–1.0, 1.5–2.0, and 2.5–3.0 strata, respectively. BSC indicates best supportive care; Local, local therapy only; Systemic, systemic therapy only; Combined, systemic plus local therapy. Tick marks denote censoring; numbers below each panel indicate patients at risk. *p‐*Values are from two‐sided log‐rank tests.

To further explore the heterogeneity of prognostic factors across different risk levels, we conducted subgroup analyses stratified by Lung‐molGPA score (0–1, 1.5–2, and 2.5–3). The results of both univariate and multivariate Cox regression analyses revealed distinct patterns of associations between clinical/treatment variables and OS within each subgroup. Notably, the survival benefit of systemic therapies (e.g., chemotherapy, immunotherapy, and antiangiogenic therapy) and local treatments (e.g., brain and chest radiotherapy/surgery) varied significantly according to Lung‐molGPA strata. A detailed summary of these subgroup‐specific hazard ratios is provided in Table 3.

**TABLE 3:** Survival impact of clinical and treatment factors across lung‐molGPA‐stratified subgroups in non‐small cell lung cancer (NSCLC) with brain metastases.

	Lung‐molGPA = 1				Lung‐molGPA = 2				Lung‐molGPA = 3			
	**Univariate analysis**		**Multivariate analyses**		**Univariate analysis**		**Multivariate analyses**		**Univariate analysis**		**Multivariate analyses**	
	**HR (95% CI)**	***p*‐Value**	**HR (95% CI)**	***p*‐Value**	**HR (95% CI)**	***p*‐Value**	**HR (95% CI)**	***p*‐Value**	**HR (95% CI)**	***p*‐Value**	**HR (95% CI)**	***p*‐Value**
Gender												
Female	1.00 (reference)		NA		1.00 (reference)		NA		1.00 (reference)		NA	
Male	1.25 (0.99–1.57)	0.060	NA		1.21 (0.93–1.58)	0.152	NA		1.06 (0.68–1.63)	0.803	NA	
Age												
0<70	1.00 (reference)		NA		1.00 (reference)		NA		1.00 (reference)		NA	
1≥70	1.09 (0.86–1.39)	0.462	NA		1.20 (0.83–1.71)	0.322	NA		1.38 (0.19–9.9)	0.752	NA	
Smoking History												
Absent	1.00 (reference)		1.00 (reference)		1.00 (reference)		NA		1.00 (reference)		NA	
Present	1.29 (1.05–1.59)	0.015*	1.34 (1.08–1.67)	0.007**	1.22 (0.98–1.53)	0.081	NA		1.29 (0.87–1.90)	0.205	NA	
Pathological												
Adenocarcinoma	1.00 (reference)		1.00 (reference)		1.00 (reference)		1.00 (reference)		1.00 (reference)		NA	
Squamous cell carcinoma	1.51 (1.12–2.05)	0.007**	1.28 (0.94–1.74)	0.118	1.57 (1.15–2.15)	0.005**	1.04 (0.76–1.45)	0.780	1.42 (0.88–2.29)	0.147	NA	
Other	1.39 (1.07–1.82)	0.015*	1.22 (0.93–1.60)	0.147	1.95 (1.43–2.65)	<0.001***	1.22 (0.88–1.69)	0.233	1.83 (0.91–3.70)	0.091	NA	
Chemotherapy cycles												
Absent	1.00 (reference)		1.00 (reference)		1.00 (reference)		1.00 (reference)		1.00 (reference)		1.00 (reference)	
1‐3	0.59 (0.46–0.76)	<0.001***	0.68 (0.53–0.88)	0.003**	0.43 (0.33–0.58)	<0.001***	0.48 (0.36–0.65)	<0.001***	0.65 (0.37–1.12)	0.123	0.48 (0.27–0.86)	0.013*
≥4	0.28 (0.17–0.31)	<0.001***	0.32 (0.23–0.45)	<0.001***	0.14 (0.10–0.20)	<0.001***	0.18 (0.12–0.26)	<0.001***	0.19 (0.11–0.33)	<0.001***	0.21 (0.12–0.37)	<0.001***
Immunotherapy												
Absent	1.00 (reference)		1.00 (reference)		1.00 (reference)		1.00 (reference)		1.00 (reference)		1.00 (reference)	
Present	0.50 (0.35–0.71)	<0.001***	0.72 (0.50–1.05)	0.092	0.36 (0.25–0.52)	<0.001***	0.60 (0.39–0.91)	0.016*	0.44 (0.24–0.79)	0.006**	0.42 (0.23–0.77)	0.004**
Antiangiogenic therapy												
Absent	1.00 (reference)		1.00 (reference)		1.00 (reference)		1.00 (reference)		1.00 (reference)		1.00 (reference)	
Present	0.50 (0.36–0.70)	<0.001***	0.73 (0.51–1.04)	0.080	0.35 (0.26–0.48)	<0.001***	0.74 (0.51–1.06)	0.100	0.40 (0.25–0.64)	<0.001***	0.52 (0.31–0.86)	0.011*
Brain radiotherapy/surgery												
Absent	1.00 (reference)		1.00 (reference)		1.00 (reference)		1.00 (reference)		1.00 (reference)		NA	
Present	0.38 (0.30–0.47)	<0.001***	0.45 (0.35–0.57)	<0.001***	0.65 (0.52–0.81)	<0.001***	0.83 (0.65–1.06)	0.144	0.96 (0.63–1.46)	0.854	NA	
Chest radiotherapy/surgery												
Absent	1.00 (reference)		1.00 (reference)		1.00 (reference)		1.00 (reference)		1.00 (reference)		1.00 (reference)	
Present	0.56 (0.39–0.80)	<0.001***	0.94 (0.65–1.37)	0.759	0.51 (0.38–0.70)	<0.001***	0.52 (0.37–0.73)	<0.001***	0.42 (0.27–0.64)	<0.001***	0.40 (0.26‐0.62)	<0.001***

To further evaluate the prognostic impact of immunotherapy, we conducted Kaplan–Meier survival analyses stratified by Lung‐molGPA score (Figures 5A–C), in addition to the analysis of the overall cohort. Among patients with low Lung‐molGPA scores (Lung‐molGPA = 0–1, Figure 5A), immunotherapy was associated with a substantial survival benefit, with mOS extended by approximately 5 months compared to the non‐immunotherapy group (p < 0.0001, log‐rank test). A similar improvement was observed in the intermediate‐risk subgroup (Lung‐molGPA = 1.5–2, Figure 5B), where the immunotherapy group exhibited significantly prolonged survival (mOS: 22.4 vs. 7.0 months; *p* < 0.0001). In the high Lung‐molGPA subgroup (Lung‐molGPA = 2.5–3, Figure 5C), immunotherapy remained associated with longer survival (mOS: 32.2 vs. 13.7 months), and the difference was statistically significant (*p* = 0.005).

**FIGURE 5:**
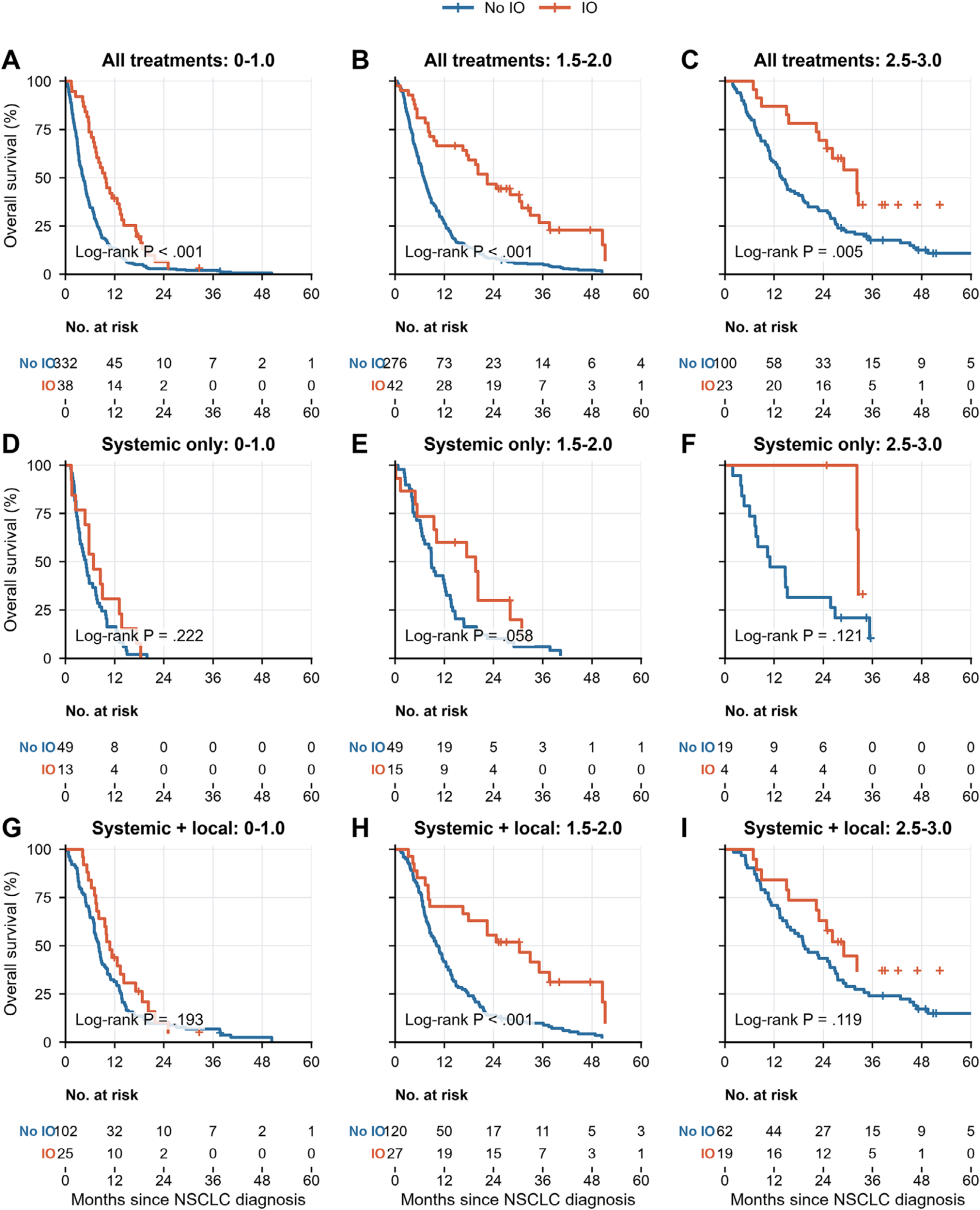
Kaplan‐Meier estimates of overall survival by immunotherapy exposure within Lung‐molGPA strata and treatment context. (A–C) All treatment contexts in the Lung‐molGPA 0–1.0, 1.5‐2.0, and 2.5‐3.0 strata, respectively. (D–F) Patients receiving systemic therapy only in the corresponding strata. (G–I) Patients receiving systemic plus local therapy in the corresponding strata. No IO indicates no immunotherapy; IO indicates immunotherapy. Tick marks denote censoring; numbers below each panel indicate patients at risk. *p‐*Values are from two‐sided, unadjusted log‐rank tests.

To further investigate the survival benefit of immunotherapy, we compared OS among patients receiving immunotherapy in combination with systemic and local therapy versus those receiving only systemic and local therapy, stratified by Lung‐molGPA score groups (Figures 5D–I).

Among patients receiving systemic therapy only (Figures 5D–F), mOS was numerically longer with immunotherapy in the Lung‐molGPA 0–1.0 stratum (6.8 vs. 4.9 months; *n* = 62; *p* = 0.222), the 1.5‐2.0 stratum (19.6 vs. 8.8 months; *n* = 64; *p* = 0.058), and the 2.5‐3.0 stratum (32.5 vs. 10.9 months; *n* = 23; *p* = 0.121). None of these unadjusted log‐rank comparisons reached statistical significance.

Among patients receiving systemic plus local therapy (Figures 5G–I), immunotherapy was not associated with a statistically significant OS difference in the Lung‐molGPA 0–1.0 stratum (10.8 vs. 8.1 months; *n* = 127; *p* = 0.193) or the 2.5‐3.0 stratum (29.0 vs. 19.1 months; *n* = 81; *p* = 0.119). In the 1.5‐2.0 stratum, mOS was longer with immunotherapy (30.3 vs. 10.1 months; *n* = 147; *p* < 0.001). Thus, statistically significant curve separation in this treatment‐context analysis was observed only in the intermediate Lung‐molGPA stratum.

## 3 Discussion

The findings from this study underscore the clinical utility of the Lung‐molGPA system not only as a prognostic stratification tool but also as a potential framework for tailoring treatment intensity and modality in patients with NSCLC and BMs who are ineligible for targeted therapies [3, 4, 6, 7]. Consistent with prior validations of the Lung‐molGPA model, higher Lung‐molGPA scores were independently associated with improved OS, reaffirming its relevance in the molecularly unselected population [7]. However, beyond mere prognostication, our data suggest that Lung‐molGPA stratification may serve as a surrogate to guide therapeutic decision‐making, particularly in delineating the threshold at which aggressive treatment confers meaningful clinical benefit.

The observed survival benefit associated with increased chemotherapy cycles, immunotherapy, antiangiogenic therapy, and localized interventions reflects the additive effect of multimodal therapy, particularly in patients with favorable prognostic features. For example, patients in the highest Lung‐molGPA category (2.5–3.0) exhibited significantly greater utilization of intensive treatment approaches, including combined systemic and local therapy, which translated into superior survival outcomes. Notably, these survival gains were not uniformly distributed across Lung‐molGPA strata. In lower Lung‐molGPA groups, the impact of treatment was attenuated, and in some cases, certain modalities such as systemic therapy alone were not significantly better than supportive care. This nuanced therapeutic responsiveness emphasizes the need for selective intensification of therapy based on prognosis and functional reserve [15].

Traditional prognostic models for brain metastases, such as RPA and GPA, were primarily developed before the widespread use of molecular targeted therapies and therefore did not incorporate key molecular biomarkers. As a result, their predictive performance in contemporary NSCLC populations is relatively limited. Previous comparative studies have reported relatively modest discrimination for these traditional models. For example, the C‐index of the original GPA and RPA models has been reported to be approximately **0.514 and 0.517**, respectively [16]. In contrast, models incorporating molecular information have demonstrated improved predictive performance. In particular, ROC‐based comparative analyses have shown that **Lung‐molGPA achieved an AUC of 0.641 for predicting 1‐year survival**, outperforming **DS‐GPA (0.572)** and **RPA (0.565)** [17]. Similarly, the reported C‐index values were **0.613 for Lung‐molGPA**, **0.569 for DS‐GPA**, and **0.603 for RPA**, indicating improved prognostic discrimination when molecular markers are incorporated. Consistent with these findings, our real‐world cohort also demonstrated reasonable predictive performance of Lung‐molGPA, with a C‐index of **0.626** and time‐dependent AUCs of **0.693, 0.765, and 0.720** for predicting 1‐, 2‐, and 3‐year OS, respectively (Table S2 and Figure S1). However, the predictive performance observed in real‐world cohorts tends to be slightly lower than that reported in model development studies, which may be attributable to patient heterogeneity, treatment evolution, and differences in molecular testing availability.

The Kaplan–Meier survival analysis further reinforces this perspective, revealing that the incremental benefit of combined systemic and local therapy was most pronounced in the higher Lung‐molGPA cohorts. In contrast, for patients with Lung‐molGPA ≤1, the lack of significant difference between local and systemic therapy highlights a potential therapeutic plateau, where survival gains are limited regardless of treatment intensification. These subgroup‐specific patterns underscore the importance of personalizing therapy not only based on molecular markers but also on validated clinical prognostic indices.

A recent Chinese cost‐of‐illness study reported that the mean total cost per patient with advanced NSCLC was approximately ¥70,000, of which 89.02% were direct costs, primarily driven by drug expenditures (approximately ¥28,000 on average), laboratory tests, and imaging procedures. Indirect (4.29%) and intangible (6.69%) costs also contributed meaningfully to the overall burden [18]. Given that patients with higher Lung‐molGPA scores in our study received more aggressive and comprehensive treatment regimens, assessing the economic impact of these approaches is essential for balancing clinical benefit with healthcare sustainability [19, 20, 21]. Notably, systemic treatments—including chemotherapy, immunotherapy, and antiangiogenic agents—demonstrated a dose‐dependent or additive survival effect, particularly in intermediate and high Lung‐molGPA groups. Immunotherapy showed pronounced benefit in Lung‐molGPA 1.5–2.0, whereas its impact diminished in Lung‐molGPA 0–1.0 and plateaued in Lung‐molGPA 2.5–3.0, suggesting a window of maximum therapeutic efficiency. Similar Lung‐molGPA‐dependent patterns were observed for antiangiogenic therapy and local chest‐directed interventions. Brain radiotherapy, while beneficial in lower Lung‐molGPA patients, offered no additional survival gain in the highest Lung‐molGPA subgroup, potentially due to a ceiling effect in patients with inherently favorable biology.

The high‐score subgroup analyses in Figure 5F,I should be interpreted cautiously. In the systemic‐therapy‐only subgroup, only four of 23 patients received immunotherapy. mOS was numerically longer with immunotherapy (32.5 vs. 10.9 months), but the log‐rank comparison was not statistically significant (*p* = 0.121). In the systemic‐plus‐local‐therapy subgroup, 19 of 81 patients received immunotherapy. mOS was 29.0 versus 19.1 months, and this comparison was also not statistically significant (*p* = 0.119). A Cox model restricted to 104 high‐score patients found no significant immunotherapy‐by‐treatment‐context interaction (HR, 2.80; 95% CI, 0.48‐16.47; *p* = 0.254). Collectively, these analyses do not show that local therapy modifies the association between immunotherapy and OS. Radiotherapy remains a biologically plausible complement to immune‐checkpoint inhibition because it can increase tumor‐antigen release and promote antitumor T‐cell responses [22]. The randomized phase 2 PEMBRO‐RT trial and a pooled analysis of two randomized trials suggested that radiotherapy may enhance out‐of‐field response and survival with pembrolizumab, although confirmatory phase 3 evidence is lacking [23, 24]. Retrospective data in patients with NSCLC brain metastases also associated concurrent stereotactic radiosurgery and PD‐1 inhibition with improved intracranial control and survival [25]. However, local treatment in our cohort was not randomized and included radiotherapy or surgery, while treatment timing was unavailable. The small and imbalanced exposed groups, wide uncertainty, selection bias, and residual confounding preclude a causal interpretation. These findings should therefore be considered hypothesis‐generating and require prospective validation.

Additionally, it must be emphasized that a formal cost‐effectiveness analysis was not performed in this study. Due to the retrospective nature of the cohort, we did not have access to the prospective quality‐of‐life assessments needed to calculate quality‐adjusted life years, nor did we possess the comprehensive individual billing records required to compute incremental cost‐effectiveness ratios. Consequently, our discussion regarding the economic implications of intensive treatments serves merely as a qualitative observation. These preliminary findings highlight the necessity for future, dedicated health economic evaluations and prospective trials to rigorously validate the cost‐effectiveness of integrating novel systemic agents and local radiotherapy across specific prognostic strata.

Finally, several limitations of this study should be acknowledged. Because our cohort was enrolled over a nearly 10‐year span (2013–2022), the utilization rates of immunotherapy (13%) and antiangiogenic therapy (17%) are relatively low compared to contemporary clinical paradigms. This reflects a transitional real‐world era rather than current standard‐of‐care patterns. However, rather than mirroring present‐day algorithms, our primary objective was to evaluate the incremental survival benefits of these newer modalities compared to conventional therapies across different Lung‐molGPA strata. Furthermore, PD‐L1 status was unavailable in 84.5% of patients, precluding adjustment for this biomarker. Consequently, the observed association between immunotherapy and OS may be affected by residual confounding related to PD‐L1 expression and treatment selection. These findings cannot establish PD‐L1‐independent immunotherapy benefit or identify which Lung‐molGPA subgroup benefits most from immunotherapy.

## 4 Conclusion

Lung‐molGPA was associated with OS in patients with EGFR/ALK/ROS1‐wild‐type NSCLC and brain metastases. Treatment–survival associations varied across prognostic strata. Tailoring treatment intensity based on Lung‐molGPA may help optimize clinical treatment strategy.

## 5 Methods

### 5.1 Data Sources, Variable Assessment, Missing Data, and Follow‐up

Clinical data were retrospectively extracted from the institutional databases and electronic medical records of the two participating centers, including demographic, pathological, molecular, imaging, treatment, and survival information. Variables were harmonized across the two centers using the same operational definitions and coding rules to improve comparability.

Eligibility for the final analytic cohort required sufficient information to confirm study eligibility, determine EGFR/ALK/ROS1 status, calculate the Lung‐molGPA score, classify treatment exposure, and ascertain OS. Patients with insufficient information for any of these core variables, including those without adequate survival information, were excluded during cohort construction. In the final analytic dataset, center, sex, age, KPS, number of brain metastases, extracranial metastasis status, smoking history, histology, Lung‐molGPA score and stratum, treatment variables, and survival time and event status each had no missing values (0/811, 0%). No statistical imputation or sensitivity analyses were performed. Because PD‐L1 status was unavailable for most patients, it was reported descriptively but excluded from the multivariable analyses.

Vital status and survival time were ascertained using institutional electronic medical records and follow‐up records. The final analytic cohort contained 769 recorded deaths and 42 right‐censored observations. Patients without a recorded death were censored on the date they were last confirmed alive. No patients were lost to follow‐up (0/811, 0%). Patients with follow‐up of 3 months or less were retained, with follow‐up recorded to the last contact and right‐censoring at that time if no death had occurred. The mean observed duration was 11.29 months, the median follow‐up estimated using the reverse Kaplan–Meier method was 51.5 months (95% CI, 46.9–65.1), and the total observation time was 762.8 person‐years.

Several potential sources of bias were considered. Requiring complete core variables and ascertainable survival information may have introduced selection bias. Because treatment was not randomly assigned, the estimated treatment associations may also have been affected by confounding by indication. Differences between the two participating centers were addressed using center‐stratified multivariable Cox regression. Nevertheless, residual confounding, center‐specific practice patterns, and temporal changes in treatment availability and clinical practice during the 2013–2022 enrollment period could not be completely eliminated.

### 5.2 Treatment Strategies

First‐line systemic therapies were administered in accordance with the CSCO and NCCN clinical guidelines for NSCLC management [26, 27]. Subsequent‐line systemic and local treatments were selected at the discretion of the treating physician, based on individual patient characteristics and disease progression. Treatment modalities included platinum‐based doublet chemotherapy or single‐agent regimens, immune checkpoint inhibitors such as pembrolizumab and tislelizumab, and antiangiogenic agents including bevacizumab and anlotinib. For brain metastases, hypofractionated radiotherapy delivered 20–32 Gy in 1–8 fractions. Conventional fractionation administered 30–54 Gy over 10–25 fractions. Chest RT was delivered at 30–60 Gy over 10–30 fractions.

### 5.3 Statistical Analysis

Univariable and multivariable Cox proportional‐hazards models were used to estimate HRs and 95% CIs for OS. Variables with univariable *p* < 0.05 were entered into the multivariable model; for variables with more than two categories, entry was based on the global likelihood‐ratio test. The center‐stratified multivariable model included Lung‐molGPA group, smoking history, pathology, chemotherapy cycles, immunotherapy, antiangiogenic therapy, brain‐directed local therapy, and chest‐directed local therapy. Low Lung‐molGPA (0–1.0) was the reference group. For Figure 2, all three Lung‐molGPA categories were retained, and the plotted coefficient represents the adjusted comparison of the high‐score group with the low‐score group. Subgroup models used the same adjustment variables, with the stratifying variable omitted from its corresponding subgroup model. Global score‐by‐subgroup interactions were evaluated using likelihood‐ratio tests. The proportional‐hazards assumption was assessed using scaled Schoenfeld residuals. Analyses were performed in R version 4.5.3 using the survival package.

## Author Contributions

B.F. and D.Y. contributed equally to this work. B.F. and D.Y. conceptualized the study, performed the statistical analysis, and drafted the initial manuscript. X.Y. and H.L. contributed to the clinical data collection, patient follow‐up, and data curation. T.W., L.C., and L.T. assisted in the validation of the prognostic models and the preparation of figures and tables. L.D. and W.Z. provided essential clinical expertise and contributed to the interpretation of the data. Y.W., Y.W., and F.J. supervised the project, secured funding, and critically revised the manuscript for important intellectual content. All authors have read and approved the final manuscript.

## Funding

This work was supported by the National Key R&D Program of China (Grant No. 2022YFC2404604); the National Natural Science Foundation of China (Grant Nos. 82073347 and 82303673); the Chongqing Municipal Talent Program “Lump‐sum Project” (Science and Technology Bureau) (Grant No. cstc2024ycjh‐bgzxm0119); the Chongqing Natural Science Foundation Project (Grant No. CSTB2024NSCQ‐KJFZMSX0056); the Chongqing Research Institution Performance Incentive Guidance Special Project (Grant Nos. CSTB2023JXL‐YFX0080 and CSTB2025JXJL‐YFX0003); the Chongqing Science and Health Joint Medical Research Project (Grant No. 2023GGXM002); the Chongqing Talent Plan (Grant Nos. CQYC20210203119 and cstc2022ycjh‐bgzxm0208).

## Conflicts of Interest

The authors declare no conflicts of interest.

## Ethics Statement

This study was conducted in accordance with the principles of the Declaration of Helsinki. The study protocol was reviewed and approved by the Ethics Committee of Chongqing University Cancer Hospital (Approval No. CZLS2023328‐A). This retrospective study was exempt from the requirement for informed consent by the Ethics Committee.

## Supporting information




**Supporting File**: mco271054‐sup‐0001‐SuppMat.docx.

## Data Availability

The datasets generated and/or analyzed during the current study are not publicly available due to institutional data protection policies but are available from the corresponding author upon reasonable request. Researchers who wish to access the raw data may contact fengbin77@126.com.
