# Supplementary material for: Optimizing Lung‐molGPA‐Directed Therapy for EGFR/ALK/ROS1‐Wildtype Non‐Small Cell Lung Cancer Brain Metastases: Survival Outcomes Analysis
Source: MedComm (2020). 2026 Oct 11;7(10):e71054. doi: 10.1002/mco2.71054 (PMC13655364; doi:10.1002/mco2.71054)
Supplement: Supplementary file 1 [file MCO2-7-e71054-s001.docx]

**Optimizing Lung-molGPA-Directed Therapy for EGFR/ALK/ROS1-Wildtype NSCLC Brain Metastases: Survival Outcomes Analysis**

Author: Bin Feng (MS)*, Dingyi Yang (M.D)*, Xin Yang (MS), Huanli Luo (Ph.D), Tian Wang (MS), Liyuan Chen(MS), Liang Tan(MS), Liang Du (M.D), Yongzhong Wu (M.D), Wei Zhou (M.D), Ying Wang (M.D), Fu Jin (Ph.D)

**Authors’ Affiliation:**

Department of Radiation Oncology, Chongqing University Cancer Hospital, Chongqing, People’s Republic of China.

***These authors contributed equally to this work.**

**Word count of the manuscript text: 5714.**

**Corresponding Authors:**

Wei Zhou, M.D. Department of Radiation Oncology, Chongqing University Cancer Hospital, No. 181 Hanyu Road, Shapingba District, Chongqing, China, 400030. e-mail: zhouwei978978@163.com.

Ying Wang, M.D. Department of Radiation Oncology, Chongqing University Cancer Hospital, No. 181 Hanyu Road, Shapingba District, Chongqing, China, 400030; e–mail: [wangying1967_cq@126.com](mailto:wangying1967_cq@126.com).

Fu Jin, Ph.D. Department of Radiation Oncology, Chongqing University Cancer Hospital, No. 181 Hanyu Road, Shapingba District, Chongqing, China, 400030. e–mail: jfazj@126.com.

Figure S1 the time-dependent ROC analysis

In our real-world cohort, the predictive performance of Lung-molGPA was also evaluated. **The Harrell’s C-index was 0.626**, and the time-dependent ROC analysis showed **AUC values of 0.693, 0.765, and 0.720** for predicting 1-, 2-, and 3-year overall survival, respectively. These findings are broadly consistent with previous validation studies and further support the clinical utility of the Lung-molGPA model in real-world populations.

Table S1 Results of the univariate cox proportional hazards model

| Variables | β | S.E | Z | *P* | HR (95%CI) |  |
| --- | --- | --- | --- | --- | --- | --- |
|  |  |  |  |  |  |  |
| Group |  |  |  |  |  |  |
| 0-1 brain therapy |  |  |  |  | 1.00 (Reference) |  |
| 0-1 no brain therapy | 1.01 | 0.11 | 8.95 | **<.001** | 2.75 (2.20 ~ 3.43) |  |
| 2.5-3 brain therapy | -0.92 | 0.15 | -6.14 | **<.001** | 0.40 (0.30 ~ 0.53) |  |
| 2.5-3 no brain therapy | -0.87 | 0.20 | -4.45 | **<.001** | 0.42 (0.28 ~ 0.61) |  |

Table S2 Comparison of predictive performance among different prognostic models for NSCLC patients with brain metastases.

| **Model** | **C-index** | **AUC (1-year OS)** |
| --- | --- | --- |
| RPA | 0.603 | 0.565 |
| DS-GPA | 0.569 | 0.572 |
| Lung-molGPA | 0.613 | 0.641 |
| Our cohort | 0.626 | 0.693 |
